# Resolvin D2 and Its Effects on the Intestinal Mucosa of Crohn’s Disease Patients: A Promising Immune Modulation Therapeutic Target

**DOI:** 10.3390/ijms26136003

**Published:** 2025-06-23

**Authors:** Livia Bitencourt Pascoal, Bruno Lima Rodrigues, Guilherme Augusto da Silva Nogueira, Maria de Lourdes Setsuko Ayrizono, Priscilla de Sene Portel Oliveira, Licio Augusto Velloso, Raquel Franco Leal

**Affiliations:** 1 Inflammatory Bowel Disease Research Laboratory, Colorectal Surgery Unit, Gastrocenter, School of Medical Sciences, University of Campinas (Unicamp), São Paulo 13083-878, Brazil; 2 Laboratory of Cell Signaling, Obesity and Comorbidities Research Center, School of Medical Sciences, University of Campinas (Unicamp), São Paulo 13083-878, Brazil

**Keywords:** Crohn’s disease, pro-resolving lipid mediator, resolvin D2, immunoregulation

## Abstract

Crohn’s disease (CD) is a chronic inflammatory disorder of the gastrointestinal tract that severely impacts patients’ quality of life. Although current therapies have improved symptom management, they often fail to alter disease progression and are associated with immunosuppressive side effects. This study evaluated the immunomodulatory potential of resolvin D2 (RvD2), a pro-resolving lipid mediator, using a murine model of colitis and the ex vivo treatment of intestinal mucosal biopsies from CD patients, comparing its effects to those of conventional anti-TNFα therapy. To determine the optimal concentration of RvD2 for application in human tissue explant cultures, an initial in vitro assay was conducted using intestinal biopsies from mice with experimentally induced colitis. The explants were treated in vitro with varying concentrations of RvD2, and 0.1 μM emerged as an effective dose. This concentration significantly reduced the transcriptional levels of TNF-α (*p* = 0.004) and IL-6 (*p* = 0.026). Intestinal mucosal biopsies from fifteen patients with CD and seven control individuals were analyzed to validate RNA-sequencing data, which revealed dysregulation in the RvD2 biosynthetic and signaling pathways. The real-time PCR confirmed an increased expression of PLA2G7 (*p* = 0.02) and ALOX15 (*p* = 0.02), while the immunohistochemical analysis demonstrated the reduced expression of the RvD2 receptor GPR18 (*p* = 0.04) in intestinal tissues from CD patients. Subsequently, samples from eight patients with active Crohn’s disease, eight patients in remission, and six healthy controls were used for the serum analysis of RvD2 by ELISA, in vitro treatment of intestinal biopsies with RvD2 or anti-TNF, followed by transcriptional analysis, and a multiplex assay of the explant culture supernatants. The serum analysis demonstrated elevated RvD2 levels in CD patients both with active disease (*p* = 0.02) and in remission (*p* = 0.002) compared to healthy controls. The ex vivo treatment of intestinal biopsies with RvD2 decreased IL1β (*p* = 0.04) and TNFα (*p* = 0.02) transcriptional levels, comparable to anti-TNFα therapy. Additionally, multiplex cytokine profiling confirmed a reduction in pro-inflammatory cytokines, including IL-6 (*p* = 0.01), IL-21 (*p* = 0.04), and IL-22 (*p* = 0.009), in the supernatant of samples treated with RvD2. Altogether, these findings suggest that RvD2 promotes the resolution of inflammation in CD and supports its potential as a promising therapeutic strategy.

## 1. Introduction

Crohn’s disease (CD) is a chronic inflammatory condition of the gastrointestinal tract that impairs patients’ quality of life and is frequently associated with extraintestinal manifestations and comorbidities such as cancer, anemia, osteoporosis, and depression [1,2,3,4,5]. Its increasing global prevalence has made CD a major clinical challenge, particularly due to the limitations of current therapeutic strategies, which often rely on corticosteroids, immunomodulators, biologic agents, or small molecules. These treatments can lead to immunosuppressive complications or treatment refractoriness, ultimately increasing the likelihood that patients will require the surgical resection of affected intestinal segments at some point in their lives. Such challenges underscore the urgent need for more effective therapeutic alternatives with improved safety profiles [6,7].

In this context, developing treatments that actively promote the resolution of inflammation without exacerbating tissue injury has emerged as a promising area of investigation. Inflammation resolution is now recognized as an active process mediated by specialized lipid mediators known as pro-resolving mediators [8,9,10]. These molecules are crucial in restoring homeostasis and preventing further damage to inflamed tissue [10]. Recent research has focused on new families of resolution-phase mediators, particularly eicosapentaenoic acid (EPA) and docosahexaenoic acid (DHA)-derived resolvins, which have demonstrated significant anti-inflammatory properties [8,9,10].

Pro-resolving mediators, such as resolvins, exert their effects by activating G-protein-coupled receptors (GPCRs) on various cell types, modulating inflammatory responses, and facilitating the resolution of inflammation [11,12]. Specifically, resolvin D2 (RvD2) is biosynthesized from DHA through a tightly regulated enzymatic pathway involving the sequential action of 15-lipoxygenase (15-LOX) and 5-lipoxygenase (5-LOX). Initially, DHA is oxygenated by 15-LOX to form a 17-hydroperoxy intermediate, which 5-LOX subsequently converts into RvD2 via specific epoxide-containing intermediates. Once generated, RvD2 exerts its pro-resolving and anti-inflammatory actions primarily through binding to G-protein-coupled receptor 18 (GPR18) [12,13]. RvD2 has shown anti-inflammatory effects in experimental models of colitis by reducing neutrophil infiltration and activation, enhancing macrophage-mediated phagocytosis and clearance, decreasing body weight loss, and lowering histological colitis scores and colon shortening—both well-established markers of experimental colitis—while also upregulating the gene expression of the anti-inflammatory cytokine IL-10 [14,15,16].

However, despite these promising experimental findings, the effects of RvD2 on the intestinal mucosa of patients with CD remains unexplored. A comprehensive transcriptomic analysis based on RNA sequencing (RNA-seq) conducted by Silva et al. [17] identified differential expression of three key genes associated with RvD2 biosynthesis and signaling—PLA2G7, ALOX5AP, and GPR18—in the intestinal mucosa of CD patients compared to non-inflammatory bowel disease (IBD) controls. Dysregulation of this pathway has been extensively documented and is increasingly recognized as implicated in the pathogenesis of various inflammatory and immune-mediated disorders [18,19,20,21,22,23].

Accordingly, the present study aimed to investigate the immunomodulatory effects of RvD2 on intestinal mucosal biopsies from CD patients, elucidating its role in modulating inflammation and comparing its therapeutic potential with that of anti-TNFα therapy, which is one of the conventional treatments.

## 2. Results

The results of this study are presented in terms of three main topics: the first involved the use of an experimental colitis model to identify the optimal dose of RvD2n to be used in the assays with human biopsies; the second focused on the biological validation of RNA-sequencing (RNA-seq) analysis and the characterization of RvD2 biosynthesis and activity in CD patients; finally, in the last one, we treated intestinal biopsies from CD patients with both active disease and in remission with RvD2, comparing the effects observed with those of the anti-TNFα treatment.

### 2.1. Murine Model Outcomes: RvD2 Attenuates DSS-Induced Colonic Inflammation In Vitro

**Figure 1:**
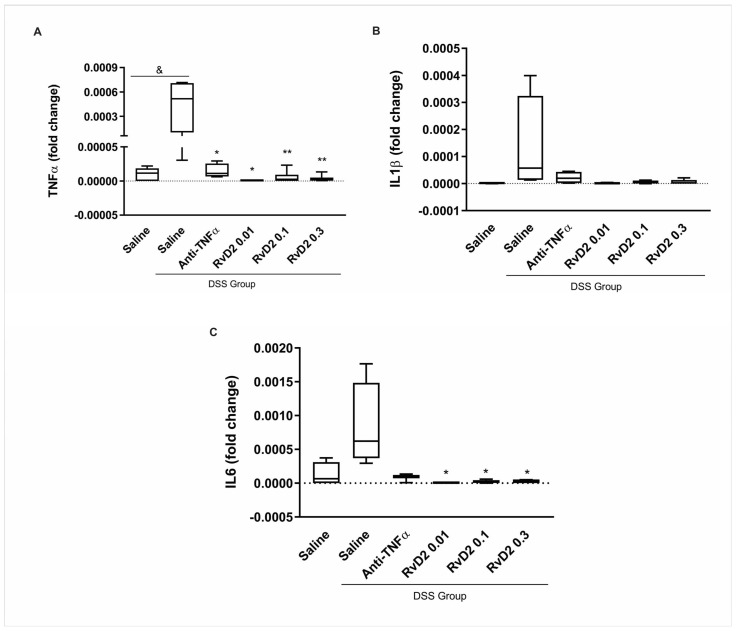
Inflammatory mediators in the intestinal mucosa of DSS-induced colitis after treatment with RvD2 and anti-TNFα in explant culture. mRNA levels (qPCR) of TNFα (**A**), IL1β (**B**), and IL6 (**C**) were measured in the intestinal mucosa of mice from the DSS group compared to the DSS-non-treated group. DSS = dextran sulfate sodium. Saline = biopsies treated with saline. Anti-TNFα = biopsies treated with infliximab at a 100 µg/mL concentration. RvD2 0.01 = biopsies treated with resolvin D2 at a concentration of 0.01 µM. RvD2 0.1 = biopsies treated with resolvin D2 at a concentration of 0.1 µM. RvD2 0.3 = biopsies treated with resolvin D2 at a concentration of 0.3 µM. *n* = 6. Data are presented as the mean ± SEM. & *p* < 0.05 was considered statistically significant compared to the DSS-non-treated group. * *p* < 0.05 and ** *p* < 0.01 were considered statistically significant compared to the saline-treated DSS group.

C57BL/6J mice were divided into the dextran sulfate sodium (DSS)-non-treated group (CTR) and DSS-induced colitis groups, with the latter receiving DSS diluted in drinking water for seven days. This treatment resulted in body weight loss, reduced food intake, and an increase in the disease activity index (DAI). Colon shortening was confirmed after euthanasia. Following this, colons were collected from both groups and cultured as explants. Three concentrations of RvD2 (0.01 µM, 0.03 µM, and 0.1 µM) were tested. The in vitro treatment of colonic biopsies from DSS-induced colitis mice with RvD2 (0.01, 0.1, and 0.3 µM) or anti-TNFα resulted in a significant downregulation of the pro-inflammatory cytokine transcripts TNFα and IL6, suggesting a marked attenuation of colonic inflammation. A significant increase in TNFα transcriptional expression was observed in saline-treated DSS biopsies compared to the DSS-non-treated group (*p* = 0.017). Within the DSS group, the treatment with anti-TNFα (*p* = 0.034), RvD2 0.01 µM (*p* = 0.015), RvD2 0.1 µM (*p* = 0.004), and RvD2 0.3 µM (*p* = 0.004) all significantly reduced TNF-α transcriptional expression when compared to the saline-treated DSS samples (Figure 1A). In contrast, IL1β expression was not significantly modulated by any treatments (Figure 1B). For IL6, although no statistically significant difference was observed in saline-treated DSS biopsies compared to the DSS-non-treated group (*p* = 0.081), the treatment with the RvD2 led to a marked reduction in transcriptional expression under RvD2 at 0.01 µM (*p* = 0.012), 0.1 µM (*p* = 0.026), or 0.3 µM (*p* = 0.026). In contrast, the anti-TNFα treatment did not significantly decrease IL6 levels compared to the saline-treated DSS group (*p* = 0.10) (Figure 1C).

These results indicate that RvD2 was effective in attenuating colonic inflammation. Furthermore, it was observed that all tested concentrations of RvD2 showed efficacy. Based on these findings, the intermediate dose of 0.1 µM of RvD2 was chosen for the subsequent stages of the study.

### 2.2. Transcriptomic and Molecular Validation of Endogenous Resolvin D2 Synthesis in Crohn’s Disease

**Figure 2:**
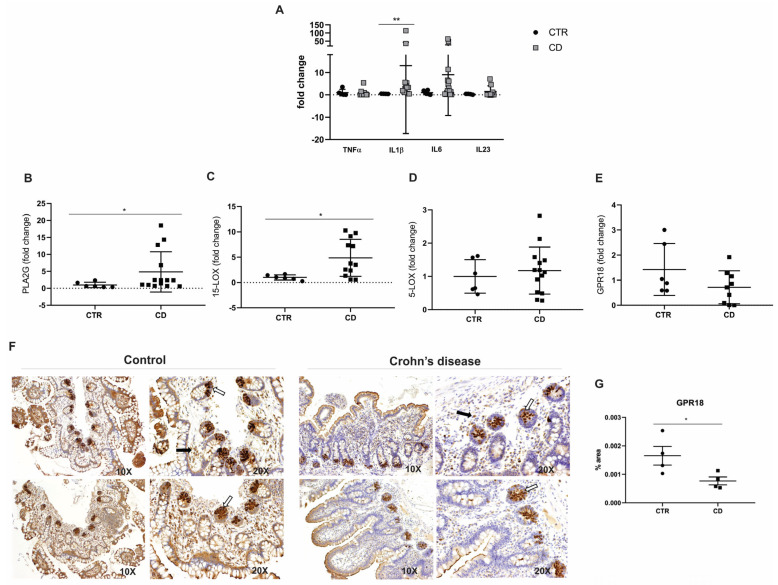
Inflammatory mediators and RvD2 biosynthesis pathway in the ileal mucosa of Crohn’s disease patients. (**A**) Transcriptional analysis of pro-inflammatory cytokines. TNFα, IL1β, IL6, and L23 mRNA levels (fold change) were measured in the ileal mucosa of Crohn’s disease patients (CD) compared to controls (CTR). (**B**–**E**) Transcriptional analysis of genes involved in the RvD2 biosynthesis pathway: (**B**) PLA2G, (**C**) 15-LOX, (**D**) 5-LOX, and (**E**) GPR18 mRNA levels (fold change) in the ileal mucosa of Crohn’s disease patients (CD) compared to controls (CTR). (**F**) Immunohistochemical analysis of GPR18 was performed on paraffin-embedded slides from the ileal mucosa of both Crohn’s disease and control groups. The white arrows indicate positive epithelial cells, and the black arrows signal positive cells from the lamina propria; 10× and 20× objective lenses were used as indicated in each image. (**G**) Quantitative analysis of RvD2 staining in the ileal mucosa of Crohn´s disease patients (CD) compared to controls (CTR). Results are presented as the mean ± SEM. CTR = control group; CD = Crohn’s disease group. *n* = 7 for CTR and *n* = 15 for CD in the transcriptional analyses; *n* = 4 for CTR and *n* = 4 for CD in the immunohistochemical analysis. * *p* < 0.05 and ** *p* < 0.01 were considered statistically significant compared to the CTR group.

The analysis of the inflammatory profile in the ileal mucosa of CD patients revealed a significant upregulation of the transcriptional levels of IL1β compared to non-IBD controls (*p* = 0.0026; Figure 2A). In contrast, the transcriptional levels of TNFα (*p* = 0.24), IL6 (*p* = 0.25), and IL23 (*p* = 0.71) did not show statistically significant differences in comparison to the control group (Figure 2A). These findings suggest a selective activation of specific pro-inflammatory pathways in the ileal mucosa of CD patients, while other cytokines remain unaffected.

To biologically validate the RNA-seq results [15], we conducted qPCR analyses, confirming a significant increase in the expression of PLA2G7 (*p* = 0.02; Figure 2B) in CD patients relative to controls. Additionally, ALOX15, a key gene involved in the biosynthesis of RvD2, was also significantly upregulated in CD patients (*p* = 0.02; Figure 2C). However, the transcriptional analysis of 5-LOX (*p* = 0.58; Figure 2D) and GPR18 (*p* = 0.12; Figure 2E) showed no statistically significant differences between the experimental groups. In addition to the transcriptional analyses, the immunohistochemical (IHC) evaluation of GPR18 protein expression—a receptor associated with RvD2 signaling—demonstrated a decrease in epithelial immunoreactivity in CD patients, especially in the base of the intestinal crypts. Fewer immunopositive cells were observed in the lamina propria of the CD group compared to controls (Figure 2F). The quantification of staining revealed a significant GPR18 reduction in protein expression in the CD group compared to the control group (*p* = 0.04; Figure 2G).

These results partially validate the RNA-seq findings and suggest a dysregulation in the biosynthesis and signaling of the pro-resolving lipid mediator RvD2 in CD patients. This dysregulation may elucidate the exacerbated inflammatory response observed in these patients.

### 2.3. Serum Levels of Resolvin D2 and Cytokine Profile After In Vitro Treatment with Resolvin D2 or Anti-TNF Therapy

**Figure 3:**
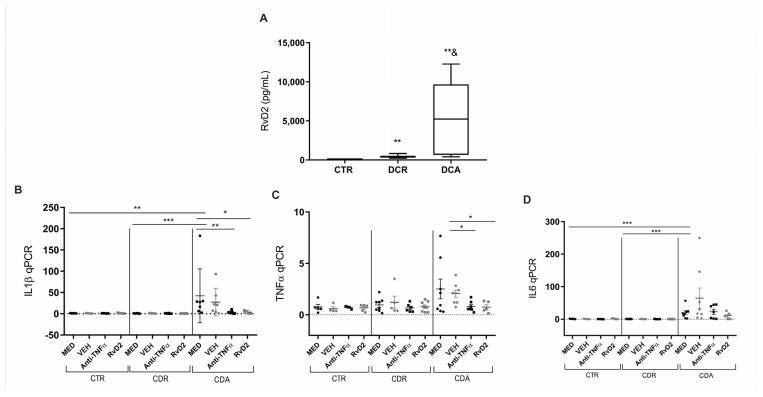
Serum resolvin D2 levels and transcriptional analysis of pro-inflammatory cytokines in Crohn’s Disease: Effects of in vitro treatment with resolvin D2 or anti-TNF therapy. (**A**) Crohn’s disease patients with active disease (CDA group) and those in remission (CDR group) exhibited increased serum levels of resolvin D2 compared to control subjects (CTR). ** *p* < 0.01 vs. CTR; *p* = 0.02 for CDA vs. CDR. (**B**–**D**) Transcriptional analysis of pro-inflammatory cytokines in Crohn’s disease following in vitro treatment with resolvin D2 or anti-TNF as an immunomodulatory agent: (**B**) IL1β transcriptional levels were significantly increased in the MED-treated CDA group compared to the MED CTR and MED CDR groups. Both anti-TNF and resolvin D2 treatments were effective in reducing IL1β expression compared to the MED CDA group; (**C**) TNFα transcriptional levels were significantly decreased in the treatment with anti-TNF or resolvin D2 conditions compared to VEH-treated CDA condition; (**D**) IL6 transcriptional levels were significantly increased in the MED CDA group compared to MED CTR and MED CDR groups; however, neither anti-TNF nor resolvin D2 treatment modulated IL6 expression compared to the MED CDA condition. CDA = Crohn’s disease in active disease. CDR = Crohn’s disease in remission. CTR = Control. MED = medium. VEH = vehicle. Anti-TNFα = infliximab. RvD2 = resolvin D2. CDA *n* = 8. CDR *n* = 8. CTR *n* = 6. * *p* < 0.05, ** *p* < 0.01 and *** *p* < 0.001 were considered statistically significant compared to the CTR group. & *p* < 0.05 were considered statistically significant compared to the CDR group.

Subsequent experiments were conducted with a new cohort of patients. Blood samples and intestinal biopsies were collected and processed in specific assays. The serum ELISA analysis demonstrated that RvD2 levels were significantly elevated in CD patients with active disease (CDA) (*p* = 0.02) as well as in those in remission (CDR) (*p* = 0.002) (Figure 3A) when compared to control patients (CTR). Additionally, RvD2 serum levels were markedly higher in the CDA group compared to the CDR group (*p* = 0.008; Figure 3A).

To further explore the inflammatory profile, a gene expression analysis was performed on biopsies from control individuals (CTR), patients with active CD (CDA), and patients with CD in remission (CDR), all treated with RvD2 or infliximab (anti-TNFα). The IL1β transcriptional level was significantly elevated in the MED-treated CDA group relative to the MED CTR (*p* = 0.01) and MED CDR (*p* = 0.0003) groups (Figure 3B). Notably, treatment with RvD2 (*p* = 0.04) and anti-TNFα (*p* = 0.007) resulted in a reduction of IL1β levels compared to the MED CDA group (Figure 3B).

Moreover, transcriptional levels of TNFα were significantly lower in the anti-TNFα (*p* = 0.01) or RvD2 (*p* = 0.02)-treated groups compared to the VEH-treated CDA group (Figure 3C). Furthermore, IL6 transcriptional levels were significantly decreased in the MED CTR (*p* = 0.0007) and MED CDR (*p* = 0.0003) groups compared to the MED CDA group (Figure 3D). However, neither the anti-TNF nor RvD2 treatment led to the modulation of IL6 transcriptional levels compared to the MED CDA condition.

These results suggest that the treatment with RvD2 reduced the inflammatory response in intestinal biopsies from patients with active CD, comparable to the effect observed in biopsies treated in culture with anti-TNFα.

### 2.4. Ex Vivo Biopsy Treatment Outcomes in Active Crohn’s Disease

**Figure 4:**
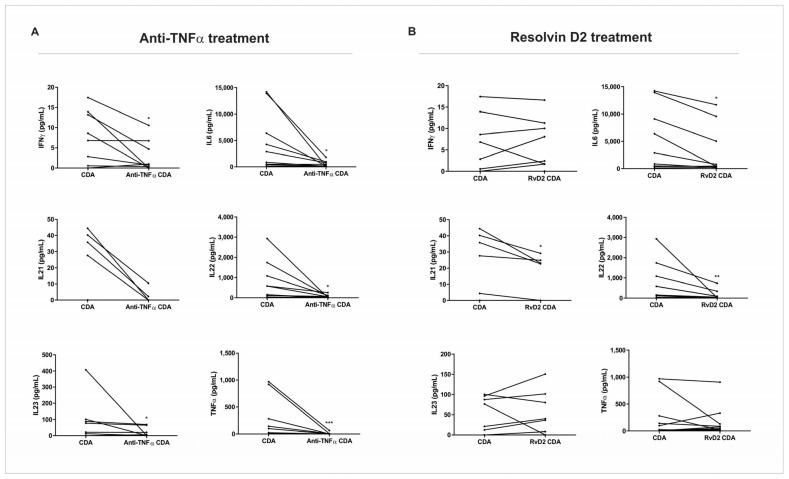
Protein expression of pro-inflammatory cytokines in supernatants of intestinal biopsy culture from active Crohn’s disease patients. Intestinal biopsies from Crohn’s disease patients in active disease (CDA group) were treated with resolving D2 (RvD2) or anti-TNFα (infliximab), and supernatants were collected for cytokine quantification using a multiplex assay. (**A**) Treatment with anti-TNFα significantly reduced the levels of IFN-γ, IL-6, IL-22, IL-23, and TNF-α compared to the respective untreated CDA group. (**B**) Treatment with resolvin D2 significantly decreased IL-6, IL-21, and IL-22 levels compared to the respective untreated CDA group. CDA = Crohn’s disease in active disease. Anti-TNFα CDA = Supernatant of intestinal biopsies culture from CDA patients treated with infliximab. RvD2 CDA = Supernatant of intestinal biopsies culture from CDA patients treated with resolvin D2. CDA *n* = 8. Anti-TNFα CDR *n* = 8. RvD2 CDA *n* = 8. * *p* < 0.05, ** *p* < 0.01 and *** *p* < 0.001 were considered statistically significant.

After the treatment with RvD2 or anti-TNFα, the supernatant of CDA was collected and used for cytokine quantification via a multiplex assay. The treatment with anti-TNFα resulted in a significant decrease in IFN-γ (*p* = 0.03), IL-6 (*p* = 0.04), IL-22 (*p* = 0.03), IL-23 (*p* = 0.04), and TNF-α (*p* = 0.001) protein expression compared to the control condition of the CDA group (Figure 4A). The treatment with RvD2 induced a significant reduction in IL-6 (*p* = 0.01), IL-21 (*p* = 0.04), and IL-22 (*p* = 0.009) protein expression when compared to the control condition of the CDA group (Figure 4B).

These findings complement the transcriptional analysis of the biopsies subjected to explant culture, confirming the attenuation of inflammation in both treatments. However, it appears that the modulation of inflammatory mediators occurs through distinct pathways.

## 3. Discussion

Crohn’s disease (CD) remains a clinical challenge, characterized by the chronic, relapsing inflammation of the gastrointestinal tract, leading to substantial morbidity and a profound impact on patients’ quality of life. While effective in achieving remission in a subset of patients, current therapeutic approaches are often hampered by significant limitations, including adverse effects, loss of efficacy over time, and the emergence of therapeutic resistance, particularly against anti-TNFα agents [24]. Therefore, identifying novel, safer, and more effective therapeutic approaches remains an unmet need. In this context, specialized pro-resolving mediators (SPMs), such as RvD2, have garnered increasing attention due to their potential to actively promote the resolution of inflammation without inducing global immunosuppression [25]. Our study sought to investigate the therapeutic potential of RvD2 in modulating intestinal inflammation in CD, with a particular focus on its comparative efficacy relative to conventional anti-TNFα therapy.

In our experimental model of colitis, RvD2 treatment consistently led to a significant reduction in the expression of key pro-inflammatory cytokines, including TNFα and IL6, across all tested concentrations (0.01 µM, 0.1 µM, and 0.3 µM). We selected the intermediate concentration (0.01 µM) for subsequent ex vivo experiments with human colonic biopsies based on the best effects and considering the risk of cytotoxicity associated with higher concentrations. This choice was made to avoid the potential lack of efficacy in patients undergoing immunosuppressive and anti-TNFα therapies, who may exhibit greater resistance to resolvin treatment. Additionally, using a higher concentration could introduce the risk of cytotoxicity, further justifying our decision to opt for the intermediate dose. These findings align with previous studies demonstrating the anti-inflammatory effects of RvD2 in experimental colitis [14,15].

The findings in the ileal mucosa of CD patients indicate a selective activation of specific pro-inflammatory pathways in the ileal mucosa of CD patients. At the same time, other cytokines, such as TNF-α, remained unaltered. Notably, TNF-α is widely recognized as being elevated in the intestinal mucosa of CD patients and is a primary target of current therapeutic strategies [26]. The absence of increased TNF-α expression in our cohort may be attributed to the clinical characteristics of the included patients, as approximately 53% were undergoing anti-TNFα therapy at the time of biopsy collection. Despite treatment, these patients exhibited active disease, suggesting that alternative inflammatory mediators, such as IL-1β, may drive persistent mucosal inflammation and contribute to therapeutic resistance. This highlights the complexity of the inflammatory milieu in CD and underscores the need for therapeutic approaches that target multiple inflammatory pathways beyond TNF-α modulation [27].

A key finding of our study was the dysregulation of genes involved in the biosynthesis and signaling of RvD2 in the intestinal mucosa of CD patients. Notably, PLA2G7 and ALOX15—critical enzymes for initiating RvD2 synthesis—were significantly upregulated; no other study had previously reported this finding, as far as we know. However, it is essential to note that these enzymes are not exclusive to RvD2 production [8,9,10]; they also contribute to the biosynthesis of other pro-resolving and pro-inflammatory lipid mediators, which may partly explain the persistence of inflammation despite elevated expression levels.

Moreover, the immunohistochemical analysis demonstrated a marked reduction in GPR18, a receptor implicated in RvD2 signaling, suggesting a compromised resolution pathway in CD. These findings, corroborated by RNA-seq data [17], support that a dysfunction of RvD2 signaling pathways may underlie the chronic inflammatory state observed in CD.

The serum analysis revealed elevated RvD2 levels in both the active and remission phases of CD compared to controls, with significantly higher concentrations during active disease. This novel observation suggests a compensatory upregulation of endogenous RvD2 in response to persistent inflammation. Nonetheless, the serum concentrations detected were substantially lower—approximately four to nineteen times lower—than those required to elicit anti-inflammatory effects in vitro. This discrepancy suggests that endogenous RvD2 production, although upregulated in serum, may be insufficient to counteract the ongoing inflammatory processes in CD, thereby reinforcing the rationale for therapeutic supplementation.

The effects of RvD2 on the pro-inflammatory cytokines were comparable to those observed with anti-TNFα therapy, suggesting that RvD2 may offer an alternative mechanism of immune modulation. Interestingly, while the anti-TNFα treatment resulted in a broader suppression of inflammatory mediators, including IFN-γ and IL-23, RvD2 exerted a more selective modulation of specific cytokine networks, mainly in patients who do not respond to anti-TNFα therapy. This differential pattern suggests that RvD2 may act through distinct receptor-mediated pathways, potentially offering a safer therapeutic profile by preserving broader immune functions. IL-21 was the only cytokine that significantly decreased in the CD group treated with RvD2 and did not in the condition treated with infliximab. IL-21 is a key player in the inflammatory processes of IBD, influencing T cell responses and Th17 development, and potentially contributing to tissue injury [28]. The role of IL-21 in promoting Th17 cell differentiation and T cell accumulation makes it a potential therapeutic target in CD, especially in anti-TNF non-responders [27]. Strategies aimed at modulating IL-21 signaling or IL-21-dependent pathways could be beneficial in reducing inflammation and promoting tissue healing [16].

Our findings align with and extend previous studies that emphasize the therapeutic potential of pro-resolving mediators in IBD [13,14,18]. Unlike traditional anti-inflammatory treatments, which primarily aim to suppress inflammation, RvD2 actively promotes the resolution of inflammation, thereby addressing a critical gap in managing chronic inflammatory disorders such as CD. The ability of RvD2 to modulate inflammation without broad immunosuppression may be particularly advantageous in maintaining host defense mechanisms during long-term treatment.

Recent translational research has provided compelling evidence for the clinical relevance of the RvD2/GPR18 axis in various health disorders [13,19]. Honkisz-Orzechowska et al. (2024) conducted a comprehensive analysis of GPR18 signaling across a range of inflammatory conditions. Their findings highlight that the action of RvD2 exerts beneficial effects in multiple pathological contexts, promoting wound healing and providing therapeutic advantages in cardiometabolic diseases, including cardiovascular disease and diabetes. Additionally, RvD2 has been associated with improvements in mild neurological conditions, including cognitive decline and mood disorders, as well as with the modulation of pulmonary diseases, such as allergies and asthma, and inflammatory joint diseases like arthritis [13].

Complementing these clinical observations, Zhao et al. (2023) elucidated the mechanistic role of the RvD2/GPR18 axis in cardiovascular and metabolic diseases, demonstrating a reduction in cerebral infarct size, attenuation of edema, and modulation of vascular inflammation. RvD2 enhances the macrophage phagocytic capacity and promotes polarization toward the anti-inflammatory M2 phenotype, which is associated with tissue repair and inflammation resolution. These effects are mediated through signaling pathways involving PKA, STAT3, and the regulation of microRNAs that suppress pro-inflammatory pathways. Although GPR18 is highly expressed in pro-inflammatory M1 macrophages, its activation by RvD2 shifts macrophage responses toward anti-inflammatory and pro-resolving states, including the suppression of the NLRP3 inflammasome. This macrophage polarization and improved phagocytosis are particularly relevant in cardiovascular disease, contributing to atherosclerosis regression and vascular protection. In hypertension, RvD2 improves vascular function by increasing nitric oxide and prostacyclin production and attenuates cardiac remodeling. During ischemia/reperfusion events such as stroke and myocardial infarction, RvD2 promotes cell survival, tissue repair, and stimulates angiogenesis and arteriogenesis. Collectively, these findings highlight the therapeutic potential of the RvD2/GPR18 axis in regulating chronic inflammation and promoting cardiovascular repair mechanisms [19].

Additionally, emerging evidence suggests that RvD2 possesses analgesic properties, which may further benefit CD patients, particularly younger individuals experiencing chronic abdominal pain and a reduced functional capacity [20,21,22]. RvD2 can improve the overall quality of life and enhance patients’ social and professional engagement by alleviating pain and reducing inflammation. Although these analgesic effects represent an important avenue for future investigation, evaluating pain-related behaviors or neuronal markers was beyond the scope of the present study. Exploring this potential in future research could yield valuable complementary insights.

Despite these promising results, some limitations of our study must be acknowledged. The small sample size for some analyses may have limited the statistical power to detect more subtle effects. These limitations largely stem from the challenges of patient recruitment and the logistical complexities involved in obtaining fresh biopsies for in vitro assays. The second cohort was prospectively recruited to meet the specific requirements of the ex vivo experimental protocol, which necessitated the immediate processing of freshly collected samples, resulting in a limited sample size. Another limitation was that the direct quantification of RvD2 in the biopsy samples via mass spectrometry (LC-MS/MS) was not performed; however, the gene expression analysis of RvD2-associated enzymes and RvD2 receptor provided meaningful insight into the altered resolution pathways in CD. Moreover, the absence of in vivo studies evaluating optimal concentrations, routes of administration, and pharmacokinetics of RvD2 administration represents a critical knowledge gap that must be addressed in future research. Ultimately, larger, multicenter studies will be crucial for validating these ex vivo findings and further evaluating the translational potential of RvD2-based interventions in clinical practice.

Importantly, our findings demonstrate that RvD2 exerts potent anti-inflammatory effects in intestinal biopsies from CD patients, comparable in magnitude to those observed with anti-TNFα therapy. Both treatments resulted in the significant downregulation of key pro-inflammatory cytokines, including IL-1β and IL-6, at the transcriptional and protein levels. However, the pattern of cytokine modulation differed: RvD2 uniquely reduced IL-21, a cytokine implicated in Th17-mediated inflammation and tissue damage, while anti-TNFα exhibited broader suppression, including effects on IFN-γ and IL-23. These results suggest that although both RvD2 and infliximab attenuate mucosal inflammation, they do so via distinct immunological pathways. RvD2 appears to engage resolution-specific mechanisms, potentially mediated through GPR18 signaling, rather than targeting upstream pro-inflammatory triggers, such as TNF-α. This divergence not only supports the therapeutic potential of RvD2 as an alternative or adjunct to current biologics but also raises the possibility of using it in patients who exhibit resistance or incomplete response to anti-TNF strategies.

## 4. Materials and Methods

### 4.1. Experimental Design and Animal Handling

Twelve male C57BL/6J mice, aged six weeks, were obtained for experimental procedures. Animals underwent a two-week acclimatization period under controlled environmental conditions (22 ± 2 °C, 12-h light/dark cycle), with unrestricted access to food and water. All experimental procedures were initiated when animals reached eight weeks of age.

### 4.2. Experimental Colitis: Induction, Validation, and Ex Vivo Biopsy Culture

**Figure 5:**
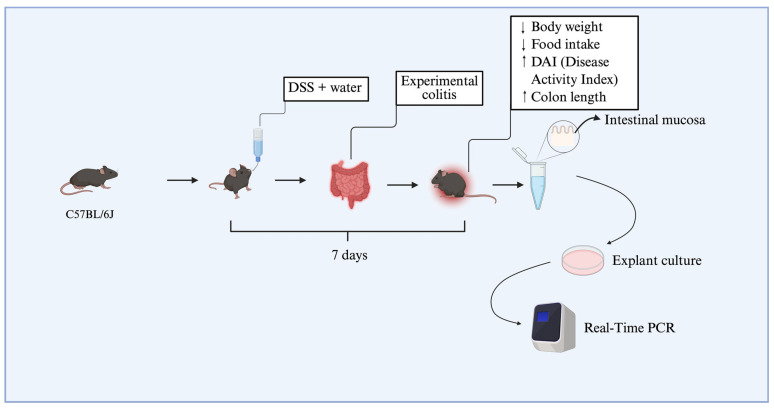
Schematic representation of the dose-response experimental design. C57BL/6J mice were divided into two groups, receiving or not receiving DSS diluted in drinking water for seven days. This resulted in weight loss, reduced food intake, and an increase in the disease activity index (DAI). Colon shortening was confirmed at euthanasia. Subsequently, colons were collected and cultured as explants, and three concentrations of resolvin D2 (0.01 μM, 0.1 μM, and 0.3 μM) were tested. The diagram illustrates the main stages of the protocol, including induction of experimental colitis with DSS, validation of methodological efficacy, tissue sample collection for explant culture, and subsequent gene expression analysis by real-time PCR. ↑: increase. ↓ decrease.

To establish dextran sulfate sodium (DSS)-induced colitis, 12 animals were allocated into two experimental groups (*n* = 6 per group), each composed of mice with a mean body weight of approximately 25 g. Dextran sulfate sodium salt (DSS), colitis grade (100 g), was purchased from MP Biomedicals (Irvine, CA, USA). The colitis group (DSS group) received 3% DSS (*w*/*v*) dissolved in drinking water for seven consecutive days, while the DSS-non-treated group received only standard drinking water. The disease activity index (DAI) was used to assess colitis induction, based on clinical parameters described in the literature, including weight loss, stool consistency, and blood in the feces or perianal region [14,15]. Daily food intake was also recorded to assess possible changes in dietary consumption associated with disease progression. At the end of the experimental protocol, animals from the DSS group that presented with an increased DAI (≥2.5), significant weight loss, and colon shortening were classified as having experimental colitis, according to previously established criteria by the research group [15]. Animals were anesthetized using thiopental and euthanized via cervical dislocation, followed by decapitation. Subsequently, colonic tissue biopsies were collected from the DSS-treated and DSS-non-treated groups. These samples were immediately processed and used in explant culture assays (Figure 5).

The collected intestinal biopsies were subsequently employed for in vitro assays using explant culture protocols (as detailed in Section 4.8). RvD2 was used at concentrations of 0.01, 0.1, or 0.3 μM, based on a previous study [29], to identify the optimal concentration for application in human tissue explant cultures. Additional DSS samples were treated with anti-TNFα (Infliximab, Remicade^®^, Janssen Biotech, Inc., Horsham, PA, USA) at a dose of 5 mg/kg, corresponding to the therapeutic dose used in humans, or with saline for the DSS-non-treated group. After treatment, the biopsies were used in real-time PCR assays (Figure 5).

### 4.3. Selection of Genes of Interest, Patient Recruitment, and Sample Collection

According to Silva et al. [17], genes associated with RvD2 were modulated in the intestinal mucosa of CD patients compared to non-IBD controls, sorted by Log2 fold change, according to the RNA-seq analysis. In this context, the transcripts phospholipase A2 (PLA2G7) and arachidonate 5-Lipoxygenase (ALOX5AP) showed a significantly increased expression in the intestinal mucosa of CD patients compared to individuals of the control group (*p* = 0.00277 and *p* = 0.04789, respectively). In contrast, GPR18, which encodes the RvD2 receptor, was significantly downregulated in CD patients relative to controls (*p* = 0.00384) (Table 1).

To biologically validate the RNA-seq findings, a first cohort of 22 individuals was enrolled, comprising 15 patients with confirmed CD and 7 individuals without IBD, who constituted the control group (CTR). The evaluation of the Crohn’s disease activity index (CDAI) was performed.

The control group (CTR) consisted of ileal biopsy samples from individuals with no history of IBD, who underwent ileocolonoscopy for screening or diagnostic purposes and presented no endoscopic or histopathological abnormalities; the biopsies were collected during the colonoscopy examination. The CD group included ileal biopsies from patients diagnosed with ileocecal CD, collected during surgical procedures (surgical specimens). In this group, mucosal samples were explicitly collected from the margins of inflamed ulcers to avoid areas containing necrotic tissue and fibrin deposits commonly present within the ulcerated regions. Immediately after collection, samples were snap-frozen and stored at −80 °C. Table 2 presents the clinical and demographic characteristics of the subjects who participated in transcriptional analysis and immunohistochemistry (first cohort).

In the second cohort, samples were collected via ileocolonoscopy from control individuals (*n* = 6), CD patients with active disease (*n* = 8), and those in remission (*n* = 8). These biopsies were promptly prepared for explant culture. The evaluation of the Crohn’s disease activity index (CDAI) was performed. Disease activity was assessed by colonoscopy (active disease defined as a CDEIS score > 5 or the presence of deep ulcers in at least one intestinal segment) or nuclear magnetic resonance (NMR) enterography (active disease defined as the presence of deep ulcers in at least one intestinal segment). Blood samples (8.5 mL) were also collected from all the groups detailed above for the performance of the ELISA assay. Laboratory analyses were performed at the IBD Research Laboratory, School of Medical Sciences, University of Campinas (Unicamp). Patients were classified according to the Montreal classification. The age of diagnosis (A) was classified as A1: diagnosis at 16 years or younger; A2: diagnosis between 17 and 40; or A3: diagnosis at 40 or older. The location of disease (L) was classified as L1: ileal involvement (small intestine); L2: colonic involvement; L3: ileocolonic involvement (both small and large intestines); or L4: upper gastrointestinal involvement. The behavior of disease (B) was classified as B1: non-stricturing, non-penetrating disease; B2: stricturing disease; or B3: penetrating disease [30]. Table 3 presents the clinical and demographic characteristics of the subjects who participated in the in vitro treatment, transcriptional analysis, and multiplex assay.

The two cohorts were designed to address different experimental objectives and were recruited independently. The first cohort, comprising samples from a biorepository, was utilized for gene expression and histological analyses. In contrast, the second cohort was recruited explicitly for ex vivo treatment and cytokine profiling, requiring freshly collected tissue to be used immediately before in vitro assays. This approach was necessary due to the time-sensitive nature of the ex vivo experiments and the logistical constraints associated with patient availability. There is no overlap of patients between the two cohorts. Each cohort consists of distinct individuals recruited independently for specific and distinct methodological and experimental purposes.

### 4.4. RNA Extraction and cDNA Synthesis

According to the manufacturer’s instructions, total RNA from intestinal mucosa samples was extracted using the RNeasy Mini Kit (Qiagen, Germantown, MD, USA). For qPCR analysis, RNA purity and concentration were determined by UV spectrophotometry at 260 nm using the BioTek Eon Microplate Spectrophotometer (BioTek Instruments, Winooski, VT, USA) and Gen5 v 2.0 software. For cDNA synthesis, the High-Capacity cDNA Reverse Transcription Kit (Applied Biosystems, Foster City, CA, USA) was used according to the manufacturer’s instructions. Afterward, the cDNA was diluted to the appropriate concentration to amplify each gene efficiently.

### 4.5. Quantitative Real-Time PCR (qPCR)

Real-time quantitative PCRs were performed using the TaqMan^TM^ system (Applied Biosystems). Primers consisted of the following: TNFa (Hs00174128_m1), IL1B (Hs_01555410), IL-6 (Hs00174131_m1), IL23 (Hs00374264), PLA2G2A (Hs00179898_m1), Alox15 (Hs00993765_g1), Alox5 (Hs00167536_m1), GPR18 (HS01921463_s1), and glyceraldehyde-3-phosphate dehydrogenase (GAPDH) (4326317E). qPCR was performed with the StepOnePlus Real-Time PCR System (Applied Biosystems, Waltham, MA, USA) using the TaqMan Fast Advanced master mix (Applied Biosystems/Life Technologies, Waltham, MA, USA). All measurements were normalized by the expression of the GAPDH gene using the delta–delta Ct method [31].

### 4.6. Immunohistochemical Staining

Intestinal mucosal samples from CD patients and control individuals were fixed in paraffin and sectioned at 4 µm thickness. Sections were subjected to deparaffinization, rehydration, and antigen retrieval using citrate buffer, followed by heating at 95 °C for 20 min. After cooling, nonspecific binding was blocked by incubation with 1% bovine serum albumin (BSA) for 45 min at room temperature.

Tissue sections were then incubated overnight at 4 °C with a primary antibody against GPR18 (1:200; NBP2-24918; Novus Biologicals, Centennial, CO, USA). The following day, slides were incubated with a biotinylated anti-rabbit secondary antibody (1:200; Vector Laboratories, CA, USA). Signal detection was performed using an avidin–biotin peroxidase complex system (Vector Laboratories), and chromogenic development was achieved with 3,3′-diaminobenzidine (DAB) substrate (Dako, A/S, Glostrup, Denmark).

After staining, sections were mounted using Dako Mounting Medium. Images were acquired using a Zeiss Axioplan 2 microscope (Carl Zeiss AG, Oberkochen, Germany) with an Olympus DP-72 digital camera and CellSens imaging software version 1.2 (Olympus Corporation, Hachioji, Japan). Representative fields were captured at 10× and 20× magnifications for subsequent analysis. Subsequently, sections were systematically scanned, and three random high-magnification fields were selected for quantitative analysis. The percentage of staining pixel volume was calculated using ImageJ2 software version 2.16.0, based on a panoramic field obtained with a 4× objective lens.

### 4.7. Enzyme-Linked Immunosorbent Assay (ELISA)

Peripheral blood samples were collected and centrifuged at 3500 rpm for 15 min at 4 °C, and the serum aliquots were snap-frozen and stored at −20 °C. The serum samples were kept at room temperature only during the ELISA analysis. Serum samples were analyzed to quantify RvD2 levels using the resolvin D2 ELISA Kit (Item No. 501120, Cayman Chemical, Ann Arbor, MI, USA).

### 4.8. Explant Culture

Intestinal mucosal biopsies were obtained from three patient groups: healthy individuals (CTR), patients with CD in remission (CDR), and patients with active CD (CDA). The samples were cultured in RPMI-1640 medium (Sigma-Aldrich^®^, Darmstadt, Germany) without L-glutamine, supplemented with 10% fetal bovine serum (FBS) and an antibiotic–antimycotic solution (Gibco, Thermo Fisher Scientific, Waltham, MA, USA).

Each biopsy was subdivided and subjected to four distinct treatment conditions: (i) control (medium only), (ii) vehicle control (medium + 0.01% ethanol, the diluent for resolvin D2), (iii) infliximab at 100 µg/mL (Remicade^®^, Janssen-Cilag, Beerse, Belgium), and (iv) resolvin D2 (RvD2) at a concentration of 0.1 µM (Item No. 10007279; Cayman Chemical Company, Ann Arbor, MI, EUA).

The selection of the 0.1 µM RvD2 dose was based on a prior dose–response analysis conducted by our group using an experimental model of DSS-induced colitis (see Figure 1). In this model, all tested RvD2 concentrations significantly reduced colonic inflammation; however, the intermediate dose (0.1 µM) was chosen for subsequent in vitro assays with Crohn’s disease patient-derived biopsies due to its consistent anti-inflammatory profile and reduced variability compared to the lower and higher doses.

Cultures were maintained for 20 h in a humidified incubator at 37 °C with 5% CO_2_. After incubation, tissue samples were collected for RNA extraction, and culture supernatants were stored for subsequent cytokine quantification.

### 4.9. Cytokines Quantification—Multiplex Assay

Following the manufacturer’s instructions, a multiplex assay was conducted using the Bio-Plex Pro Human Th17 Cytokine Assay Kit (catalog number 171AA001M, Bio-Rad Laboratories, Hercules, CA, USA). The concentrations of cytokines, including IL-1β, IL-4, IL-6, IL-10, IL-17A, IL-17F, IL-21, IL-22, IL-23, IL-25, IL-31, IL-33, CD40L, IFN-γ, and TNF-α, were quantified from the supernatants of explant cultures. The assay plates were pre-wetted and washed before coupling magnetic beads to a 96-well plate. Serial dilutions of the reconstituted standard and experimental samples were added to the wells. The mixture was incubated in the dark at room temperature, on an orbital shaker for 60 min. Following incubation, detection antibodies were diluted in antibody diluent, added to each well, and incubated for 30 min at room temperature. After the washing step, streptavidin-PE was added and incubated for 10 min at room temperature. Following a final washing step, the samples were analyzed using a Luminex system operated with Bio-Plex Manager software (version 6.0) at the Life Sciences Core Facility (LaCTAD), University of Campinas (Unicamp).

**Figure 6:**
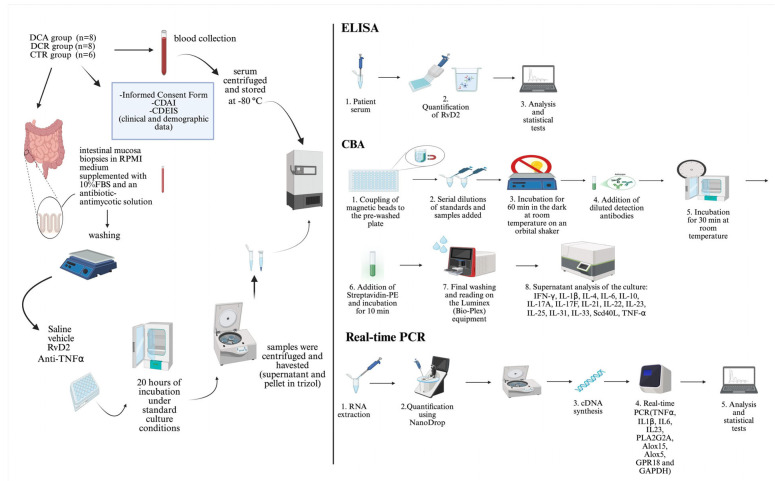
Schematic representation of the ex vivo treatment of the intestinal mucosa biopsies with resolvin D2 or anti-TNFα, and the processing of the samples. ELISA and CBA assays were employed for protein analysis, and real-time PCR was performed for transcriptional analysis.

The summary of the method applied in the ex vivo treatments is represented in Figure 6.

### 4.10. Statistical Analysis

All data were analyzed and reported using median values. The Kolmogorov–Smirnov test was used to investigate whether the data followed a normal Gaussian distribution (*p* > 0.1). The Grubbs test (https://www.graphpad.com/quickcalcs/grubbs1/, accessed on 19 March 2024) was used to identify the outliers. Once normality was established, the *t*-test and Mann–Whitney test were performed between the groups. The level of significance was set at *p* < 0.05. All analyses were performed using GraphPad Prism version 8.0.

## 5. Conclusions

This study demonstrates that RvD2 significantly reduces pro-inflammatory cytokines such as IL-1β, IL-6, and IL-21 in ex vivo intestinal biopsies from CD patients, including those refractories to anti-TNFα therapy. RvD2 appears to act independently of TNF-α signaling and may help restore resolution pathways that are disrupted in CD, as evidenced by reduced GPR18 receptor expression in patient tissues. Unlike infliximab, RvD2 selectively modulated distinct cytokine networks, suggesting a novel mechanism of immune modulation. These findings support RvD2 as a promising therapeutic candidate and underscore the need for future in vivo studies to assess its pharmacodynamics, delivery strategies, and long-term safety in human IBD.

## Figures and Tables

**Table 1:** Selected genes of interest were identified as differentially expressed in the ileal mucosa of Crohn’s disease patients (CD) compared to control (CTR) individuals, based on RNA-sequencing analysis (Ramos da Silva et al. 2020) [17].

Gene Name	Gene Symbol	Locus	Log2 Fold Change	*p* Value
Phospholipase A2 group VII	*PLA2G7*	6p21.2-p12	1.93	0.002
Arachidonate 5-lipoxygenase-activating protein	*ALOX5AP*	13q12	0.77	0.047
G protein-coupled receptor 18	*GPR18*	13q32	−1.52	0.003

For the CD group, *n* = 8; for the CTR group, *n* = 4.

**Table 2:** Clinical and demographic data of the patients included in the biological validation through transcriptional analysis and immunohistochemistry.

	CTR	CD
Number	7	15
Gender (M/F)	2/5	6/9
Age (year)	56 (44–71)	35 (20–70)
Disease duration (months)	-	95 (11–360)
Age at diagnosis (A1/A2/A3) *	-	1/12/2
Location (L1/L2/L3/L4) *	-	4/0/11/0
Behaviour (B1/B2/B3) *	-	0/8/7
Perianal disease (yes/no)	-	1/14
Immunosuppressant (yes/no)	-	5/10
Biologics (infliximab/adalimumab/none)	-	6/2/7
BMI	-	21 (14.7–30.1)
CDAI	-	319.4 (162–479.8)

Numerical variables are described as the median [min, max], and categorical variables are expressed as absolute frequencies. * Montreal classification. Disease classification followed the Montreal system: age at diagnosis (A)—A1: ≤16 years; A2: 17–40 years; A3: >40 years. Disease location (L)—L1: ileal (small intestine); L2: colonic; L3: ileocolonic (both small and large intestine); L4: isolated upper gastrointestinal involvement. Disease behavior (B)—B1: non-stricturing, non-penetrating; B2: stricturing; B3: penetrating. CD = Crohn’s disease. CTR = control. M = male. F = female. BMI = Body Mass Index. CDAI = Crohn’s disease activity index.

**Table 3:** Clinical and demographic data of the patients included in the transcriptional analysis, ELISA, and multiplex assay of the culture of explants.

	CDA Group	CDR Group	CTR Group	*p*-Value
Number	8	8	6	-
Gender (M/F)	2/6	5/3	2/4	0.416
Age (year)	48.5	57.5	53.5	0.34
Disease duration (months)	204	156	-	0.246
Age at diagnosis (A1/A2/A3) *	1/4/3	1/3/4	-	0.867
Location (L1/L2/L3/L4) *	0/2/6/0	1/0/7/0	-	0.215
Behaviour (B1/B2/B3) *	0/5/3	3/4/1	-	0.128
Perianal disease (yes/no)	4/4	2/6	-	0.302
Immunosuppressant (yes/no)	4/4	6/2	-	0.302
Biologics (yes/no)	6/2	6/2	-	1
BMI	25.89	24.35	-	0.728
CDAI	115.7	134	-	0.907
CDEIS	5.8 **	0	-	<0.001

Numerical variables are described as the median [min, max], and categorical variables are expressed as absolute frequencies. * Montreal classification. Disease classification followed the Montreal system: age at diagnosis (A)—A1: ≤16 years; A2: 17–40 years; A3: >40 years. Disease location (L)—L1: ileal (small intestine); L2: colonic; L3: ileocolonic (both small and large intestine); L4: isolated upper gastrointestinal involvement. Disease behavior (B)—B1: non-stricturing, non-penetrating; B2: stricturing; B3: penetrating. CDA = active Crohn’s disease. CDR = Crohn’s disease in remission. CTR = Control. M = male. F = female. BMI = Body Mass Index. CDAI = Crohn’s disease activity index. CDEIS = Crohn’s disease Endoscopic Index of Severity. All patients receiving biologic therapy were treated with infliximab. ** *p* < 0.01 vs. CDR group.

## Data Availability

The original contributions presented in this study are included in the article. Further inquiries can be directed to the corresponding author.

## Abbreviations

DSS	Dextran Sulfate Sodium
DAI	Disease Activity Index
RvD2	Resolvin D2
TNF-α	Tumor Necrosis Factor Alpha
IL-6	Interleukin 6
IL-1β	Interleukin 1 Beta
IL-10	Interleukin 10
IL-17A	Interleukin 17A
IL-17F	Interleukin 17F
IL-21	Interleukin 21
IL-22	Interleukin 22
IL-23	Interleukin 23
IL-25	Interleukin 25
IL-31	Interleukin 31
IL-33	Interleukin 33
IFN-γ	Interferon Gamma
qPCR	Quantitative Polymerase Chain Reaction
IHC	Immunohistochemistry
ELISA	Enzyme-Linked Immunosorbent Assay
GPCR	G-protein Coupled Receptor
EPA	Eicosapentaenoic Acid
DHA	Docosahexaenoic Acid
PLA2G7	Phospholipase A2 Group VII
ALOX5AP	Arachidonate 5-Lipoxygenase-Activating Protein
5-LOX	5-Lipoxygenase
GPR18	G-protein Coupled Receptor 18
CDA	Crohn’s Disease Active
CDR	Crohn’s Disease Remission
MED	Medium
VEH	Vehicle
RPMI-1640	Medium
FBS	Fetal Bovine Serum
CDAI	Crohn’s Disease Activity Index
CDEIS	Crohn’s Disease Endoscopic Index of Severity
NMR	Nuclear Magnetic Resonance
RNeasy Mini Kit	RNA extraction kit by Qiagen
cDNA	Complementary Deoxyribonucleic Acid
GAPDH	Glyceraldehyde-3-Phosphate Dehydrogenase
DAB	3,3′-Diaminobenzidine
IBD	Inflammatory Bowel Disease
CD	Crohn’s Disease
FAPESP	Fundação de Amparo à Pesquisa do Estado de São Paulo (São Paulo Research Foundation)
FAEPEX	Funding for Education, Research and Extension Support (University of Campinas)
CAPES	Coordination for the Improvement of Higher Education Personnel (Brazil)
CEUA/UNICAMP	Animal Ethics Committee of the University of Campinas
CAAE	Research Ethics Committee Registration Number
Log2	Logarithm base 2
RNA-seq	RNA Sequencing
Bio-Plex Pro Human Th17 Cytokine Assay Kit	Multiplex assay kit for cytokine analysis
